# SARS-CoV-2 Infection and Response to COVID-19 Vaccination in Patients With Primary Immunodeficiencies

**DOI:** 10.1093/infdis/jiad145

**Published:** 2023-08-04

**Authors:** Robert Paris

**Affiliations:** Moderna, Inc., Cambridge, Massachusetts, USA; Department of Medicine, Infectious Diseases, University of Maryland School of Medicine, Baltimore, Maryland, USA

**Keywords:** COVID-19, mRNA, primary immunodeficiencies, SARS-CoV-2, vaccination

## Abstract

Primary immunodeficiencies (PIDs) are heterogeneous, rare disorders that increase susceptibility to infection and/or immune dysregulation. Individuals with certain PIDs are at high risk of severe or fatal outcomes from SARS-CoV-2 infections (the causative agent of COVID-19), either due to the underlying PID and/or due to the presence of comorbidities such as severe lung and liver disease. Vaccination remains the primary strategy to protect individuals with PID from COVID-19. However, populations with PID exhibit variable vaccine seroresponse rates, antibody titers, and neutralization activity depending on the type of PID and/or COVID-19 vaccine, and consequently, are at an elevated risk of severe disease. In this article, we review the COVID-19 burden in patients with PIDs and focus in-depth on findings from patients with predominantly antibody deficiencies or combined immunodeficiencies. We conclude by providing COVID-19 vaccination recommendations for this population.

## INDIVIDUALS WITH PRIMARY IMMUNODEFICIENCIES AND THE IMPACT OF THE COVID-19 PANDEMIC

Primary immunodeficiencies (PIDs) are heterogeneous, rare disorders in which 1 or more components of the immune system are deficient, leading to increased susceptibility to infection and/or immune dysregulation [1]. There are currently 485 single gene defects that cause PIDs [2, 3]; however, in some, the underlying genetic defects are largely unknown [2]. There is considerable genetic heterogeneity (defects in different genes can lead to a similar phenotype), variable expressivity (defects in the same gene can lead to a variable phenotype), and variable penetrance in PIDs [2]. Consequently, the clinical presentation of PIDs is broad, with the most prominent clinical features including an increased susceptibility to infection, immune dysregulation, autoimmunity, and increased propensity to malignancy [2]. Global estimates report that PIDs occur at a rate of 0.8 to 20.2 cases per 100 000 [4]; however, PIDs are frequently misdiagnosed, which suggests that this rate may be an underestimation [5].

Primary immunodeficiencies are typically grouped into defects of adaptive immunity (T cells and B cells) and innate immunity (eg, complement phagocytic defects) [2]. To aid in the diagnosis and clinical management of PIDs, the International Union of Immunological Societies has formally classified them into 10 broad categories based on underlying abnormalities: (1) combined immunodeficiencies (CIDs); (2) CIDs with syndromic features; (3) predominantly antibody deficiencies (PADs); (4) diseases of immune dysregulation; (5) congenital defects of phagocytes; (6) defects in intrinsic and innate immunity; (7) autoinflammatory diseases; (8) complement deficiencies; (9) bone marrow failure; and (10) phenocopies of PIDs [2].

It has become apparent during the COVID-19 pandemic that patients with certain PIDs are more likely to have severe or fatal infections, either due to the underlying PID and/or due to the presence of comorbidities such as severe lung and liver disease [6]. In addition, new PIDs have been discovered that may predispose an individual to severe COVID-19. These PIDs may involve defects in genes that lead to the production and/or response to type I interferons, placing these individuals at higher risk of severe COVID-19 [2].

The availability of COVID-19 vaccines, such as mRNA-1273 (SPIKEVAX; Moderna, Inc., Cambridge, MA, USA) and BNT162b2 (COMIRNATY; Pfizer Inc., New York, NY, USA; BioNTech Manufacturing GmbH, Mainz, Germany), has eased the threat of COVID-19, and vaccination remains the primary strategy to protect individuals with PIDs from COVID-19. However, the suboptimal responses to vaccination due to impaired cellular and humoral responses in these patients continues to place this population at risk of severe outcomes from disease [7–10]. As the circulation of SARS-CoV-2 becomes more endemic, it remains important to assess responses to infection and vaccination, stratified by PID classification, to better inform clinical practice. In this study, where available, we summarize the literature on COVID-19-related clinical outcomes, immune responses after SARS-CoV-2 infection, and COVID-19 vaccination in patients with PIDs. Evidence that vaccines such as mRNA-1273 and BNT162b2 can decrease the severity and mortality of disease is reviewed, and approaches to enhance vaccination outcomes are summarized to serve as a resource to guide decisions regarding COVID-19 vaccination in this population. In this review, we provide an overview of the COVID-19 burden in patients with PIDs, followed by an in-depth focus on findings from patients with PADs or CIDs.

## INDIVIDUALS WITH PRIMARY IMMUNODEFICIENCIES ARE AT HIGH RISK FOR SARS-CoV-2 INFECTIONS AND POOR OUTCOMES FROM COVID-19

Studies have consistently shown that individuals with PIDs mount poor responses to SARS-CoV-2 infection compared with immunocompetent populations; these responses also vary greatly between PID groups themselves. Several studies have reported that SARS-CoV-2 infection rates among various PID populations range from 0.7% to 63.3% (Table 1) [11, 13, 14]. In addition, despite similar rates of COVID-19-related hospitalization between various PID groups (49.0%–53.3%) [12, 14], a wide range of COVID-19 mortality rates have been reported and range from 3.8% to 42.1% [11–14]. Certain comorbidities, such as preexisting lung disease or chronic liver disease, have also been associated with higher hospitalization rates in populations with PIDs (62.5% and 12.5%, respectively) when compared with populations without PIDs (16.7% and 3.1%, respectively) [14]. It is notable that, at the start of the COVID-19 pandemic, severe outcomes after SARS-CoV-2 infection in PID populations were particularly high, with reports from small cohorts of uncategorized PID patients indicating that 63% required hospitalization due to COVID-19, with a case-fatality rate of 10% to 38%; similarly, high case-fatality rates were reported in individuals receiving immunotherapy and secondary immunodeficiencies (discussed in more detail within the second article of this supplement) [11, 14, 17, 18]. Higher mortality during the early stages of the pandemic may have been due to differences in viral strains, as well as the lack of SARS-CoV-2-specific treatments and vaccines [10].

**Table 1.** COVID-19 Burden in Patients With PIDs

Publication First Author	Disease (Population)	Morbidity and Mortality Rates		
		SARS-CoV-2 Infection, %	Hospitalization, %	Mortality, %
Heterogeneous PID				
Delavari [11]	Heterogeneous PID (N = 2754)	0.7%	NA	42.1%
Dryzmalla [12]	Heterogeneous PID (N = 459)	NA	49.0%	9.4%
Milito [13]	Heterogeneous PID (N = 3263)	4.0%	NA	3.8%
Shields [14]	Heterogeneous PID (N = 60)	63.3%	53.3%	20.0%
Combined Immunodeficiencies				
Chappel [15]	Heterogeneous CID (N = 1022)	33.7%	0.4%	0.0%
Delavari [11]	Heterogeneous CID (N = 630)	1.5%	NA	60.0%
Dryzmalla [12]	Heterogeneous CID (N = 35)	NA	38%	8.8%
Milito [13]	22q11 deletion syndrome (N = 527)	2.3%	NA	8.3%
Delavari [11]	Ataxia telangiectasia (N = 86)	1.1%	NA	0.0%
Milito [13]	Ataxia telangiectasia (N = 54)	3.7%	NA	0.0%
Milito [13]	CD4 lymphopenia (N = 26)	7.7%	NA	0.0%
Milito [13]	Hyper IgE (N = 50)	0%	NA	0.0%
Delavari [11]	Nonsyndromic CID (N = 247)	2.4%	NA	83.3%
Delavari [11]	SCID (N = 113)	4.4%	NA	80.0%
Predominantly Antibody Deficiencies				
Dryzmalla [12]	Heterogeneous PAD (N = 208)	NA	50.5%	8.4%
Delavari [11]	CVID (N = 352)	0.2%	NA	0.0%
Milito [13]	CVID (N = 1611)	6.4%	NA	4.1%
Shields [14]	CVID (N = 23)	69.6%	56.5%	34.8%
Delavari [11]	Congenital agammaglobulinemia (N = 147)	0.6%	NA	0.0%
Milito [13]	Congenital agammaglobulinemia (N = 17–148)	8.8%–17.6%	NA	0.0%
Ponsford [16]	Congenital agammaglobulinemia (N = 28)	NA	78.6%	3.6%

Abbreviations: CID, combined immunodeficiency; CVID, common variable immunodeficiency; Ig, immunoglobulin; N, total population reported within the study; NA, information is not presented in source article; PAD, predominantly antibody deficiency; PID, primary immunodeficiency; SCID, severe combined immunodeficiency.

After COVID-19 vaccines became available, early data indicated that immunologic responses and vaccine effectiveness in patients with PIDs were affected by the variability in severity and type of immunodeficiency, which has been well described for other vaccines, to the extent that assessment of vaccine response is commonly used in the evaluation and diagnosis of suspected PIDs (Table 2) [39]. Several studies demonstrated that after vaccination, different PID groups had variable seroresponse rates, antibody titers, and neutralization activity depending on the COVID-19 vaccine type, with mRNA vaccines inducing greater humoral responses compared with vector-based vaccines [8, 21]. However, vaccine-induced antibody responses alone may not necessarily correlate with prevention of COVID-19 hospitalization, because other immune mediators, such as vaccine-specific T-cell responses, also assist in preventing severe COVID-19 outcomes [40]. Results from studies investigating antibody and cellular responses after a 2-dose primary series of COVID-19 vaccination in patients with PIDs indicated that 48.5% to 86.0% of patients had binding antibodies against SARS-CoV-2 [7–9, 19, 21, 22, 27], whereas 73.1% to 87.0% of patients had T-cell responses [7, 8, 19, 21, 22, 27]. In general, a primary COVID-19 vaccine series induced substantially lower immune responses in patients with PIDs compared with healthy controls [8, 19, 22, 41]; these differences were especially profound with regards to neutralizing titers against omicron [23].

**Table 2.** COVID-19 Vaccine Responses in Patients With PIDs

Publication First Author	Disease (Population)	Vaccination Regimen	Vaccine(s)	Antibody Responses		Cellular Responses, %
				Participants With Binding Antibodies, %	Participants With Neutralizing Antibodies, %	
Heterogeneous PID						
Amodio [19]	Heterogeneous PID (N = 21)	Primary Series^a^	BNT162b2	86%	NA	76%
Delmonte [20]	Heterogeneous PID (N = 46)	Primary Series^a^	BNT162b2, mRNA-1273	85.4%	NA	Increased
Durkee-Shock [9]	Heterogeneous PID (N = 90)	Primary Series^a^	BNT162b2	73%	NA	NA
	Heterogeneous PID (N = 304)	Primary Series^a^	BNT162b2, ChAdOx1	67%	NA	NA
Erra [21]	Heterogeneous PID (N = 118)	Primary Series^a^	BBIBP-CorV, BNT162b2, ChAdOx1, mRNA-1273, Sputnik V	80.6%	81.0%	87.0%
Göschl [8]	Heterogeneous PID (N = 26)	Primary Series^a^	BNT162b2, ChAdOx1, mRNA-1273	76.9%	NA	82%
Hagin [22]	Heterogeneous PID (N = 26)	Primary Series^a^	NA	69.2%	NA	73.1%
Nadesalingam [23]	Heterogeneous PID (NA)	Primary Series^a^	BNT162b2, mRNA-1273	NA	Omicron: 17%–22%	NA
Pham [7]	Heterogeneous PID (N = 33)	Primary Series^a^	BNT162b2, mRNA-1273	48.5%	NA	77.4%
Nadesalingam [23]	Heterogeneous PID (NA)	Additional Dose	BNT162b2, mRNA-1273	NA	Omicron: 30%–40%	NA
Combined Immunodeficiencies						
Ainsua-Enrich [24]	Heterogeneous CID (N = 1)	Primary Series^a^	mRNA-1273	0%	NA	NA
Bracke [25]	Heterogeneous CID (N = 1)	Primary Series^a^	BNT162b2, mRNA-1273	0%	NA	NA
Van Leeuwen [26]	Heterogeneous CID (N = 25)	Primary Series^a^	mRNA-1273	71%	86%	NA
Delmonte [27]	Idiopathic T-cell lymphopenia (N = 11)	Primary Series^a^	NA	90.9%	NA	NA
Ainsua-Enrich [24]	Heterogeneous CID (N = 1)	Additional Dose	mRNA-1273	100%	Low	NA
Predominantly Antibody Deficiencies						
Ainsua-Enrich [24]	CVID (N = 12)	Primary Series^a^	mRNA-1273	67%	Wild type: High titers Delta: Medium titers Omicron: Low titers	Similar to healthy controls
Antoli [28]	CVID (N = 28)	Primary Series^a^	Ad26.COV2.S, BNT162b2, ChAdOx1, mRNA-1273	71.4%	NA	71%
Arroyo-Sánchez [29]	CVID (N = 18)	Primary Series^a^	BNT162b2, ChAdOx1, mRNA-1273	83%	50%	83%
Barmettler [30]	CVID (N = 21)	Primary Series^a^	Ad26.COV2.S, BNT162b2, mRNA-1273	76.2%	72.3%	NA
Bitzenhofer [31]	CVID (N = 26)	Primary Series^a^	BNT162b2, mRNA-1273	61.5%	NA	NA
Bracke [25]	CVID (N = 14)	Primary Series^a^	BNT162b2, mRNA-1273	14.3%	NA	NA
Carrabba [32]	CVID (N = 12)	Primary Series^a^	BNT162b2	50%	NA	NA
	CVID (N = 37)	Primary Series^a^	mRNA-1273	78.4%	NA	NA
Delmonte [27]	CVID (N = 6)	Primary Series^a^	NA	100%	NA	NA
	CVID (N = 8)	Primary Series^a^	NA	87.5%	NA	NA
	CVID (N = 15)	Primary Series^a^	NA	80%	NA	NA
	CVID (N = 12)	Primary Series^a^	NA	83.3%	NA	NA
	CVID (N = 15)	Primary Series^a^	NA	73.3%	NA	NA
	CVID (N = 4)	Primary Series^a^	NA	75%	NA	NA
	CVID (N = 33)	Primary Series^a^	NA	33%	NA	NA
	CVID (N = 34)	Primary Series^a^	NA	23.5%	NA	NA
	CVID (N = 38)	Primary Series^a^	NA	36.8%	NA	NA
	CVID (N = 14)	Primary Series^a^	NA	92%	NA	NA
	CVID (N = 41)	Primary Series^a^	NA	68.3%	NA	NA
	CVID (N = 60)	Primary Series^a^	NA	71.7%	NA	NA
	CVID (N = 18)	Primary Series^a^	NA	83%	50%	NA
	CVID (N = 31)	Primary Series^a^	NA	48.4%	NA	NA
Durkee-Shock [9]	CVID (N = 17)	Primary Series^a^	BNT162b2	65%	NA	NA
	CVID (N = 17)	Primary Series^a^	BNT162b2	70.5%	NA	82%
	CVID (N = 30)	Primary Series^a^	ChAdOx1 with BNT162b2 booster	83%	80%	53% after ChAdOx1 83% after BNT162b2 booster
	CVID (N = 5)	Primary Series^a^	BNT162b2, mRNA-1273	80%	NA	NA
Erra [21]	CVID (N = 59)	Primary Series^a^	BBIBP-CorV, BNT162b2, ChAdOx1, mRNA-1273, Sputnik V	78.0%	NA	NA
Gernez [33]	CVID (N = 10)	Primary Series^a^	BNT162b2, mRNA-1273	100%	NA	NA
Pham [7]	CVID (N = 15)	Primary Series^a^	BNT162b2, mRNA-1273	80%	NA	80%
Pulvirenti [34]	CVID (Convalescent + vaccinated, N = 20; vaccinated only, N = 38)	Primary Series^a^	BNT162b2	Convalescent and vaccinated: >82% Vaccinated: 34%	NA …	Convalescent and vaccinated: absent Vaccinated: 1.2%
Romano [35]	CVID (N = 5)	Primary Series^a^	BNT162b2, mRNA-1273	NA	80%	NA
Salinas [36]	CVID (N = 34)	Primary Series^a^	BNT162b2	20.6%	NA	NA
Sauerwein [37]	CVID (N = 31)	Primary Series^a^	BNT162b2	48.4%	NA	NA
Shin [38]	CVID (N = 12)	Primary Series^a^	BNT162b2, mRNA-1273	58%	NA	NA
Van Leeuwen [26]	CVID (N = 212)	Primary Series^a^	mRNA-1273	81%	72%	67%
Carrabba [32]	Congenital agammaglobulinemia (N = 6)	Primary Series^a^	BNT162b2, mRNA-1273	67%	NA	NA
Delmonte [27]	Congenital agammaglobulinemia (N = 1)	Primary Series^a^	NA	0%	NA	NA
	Congenital agammaglobulinemia (N = 2)	Primary Series^a^	NA	NA	NA	100%
	Congenital agammaglobulinemia (N = 4)	Primary Series^a^	NA	50%	NA	75%
	Congenital agammaglobulinemia (N = 4)	Primary Series^a^	NA	0%	NA	NA
	Congenital agammaglobulinemia (N = 3)	Primary Series^a^	NA	0%	NA	NA
	Congenital agammaglobulinemia (N = 7)	Primary Series^a^	NA	0%	NA	83%
Salinas [36]	Congenital agammaglobulinemia (N = 6)	Primary Series^a^	BNT162b2	0%	NA	NA
Van Leeuwen [26]	Congenital agammaglobulinemia (N = 21)	Primary Series^a^	mRNA-1273	15%	NA	NA
Ainsua-Enrich [24]	CVID (N = 12)	Additional Dose	mRNA-1273	58%	Wild-type: High titers Delta: Medium titers Omicron: Low titers	Increased
Barmettler [30]	CVID (N = 21)	Additional Dose	Ad26.COV2.S, BNT162b2, mRNA-1273	9-fold increase compared with dose 2	NA	NA
Delmonte [27]	CVID (N = 10)	Additional Dose	NA	100%	NA	NA
Sauerwein [37]	CVID (N = 31)	Additional Dose	BNT162b2	Lower than healthy controls	NA	Lower than healthy controls

Abbreviations: CID, combined immunodeficiency; CVID, common variable immunodeficiency; N, total population reported within the study; NA, information is not presented in source article; PID, primary immunodeficiency.

COVID-19 vaccine primary series are administered as either a single dose or a 2-dose regimen.

In summary, PIDs are highly heterogeneous, and, thus, there are variable outcomes in patient with PIDs from COVID-19 and vaccination. In the following sections, we review more specifically the disease burden and vaccination responses in patients with PADs and CIDs.

## POPULATIONS WITH PREDOMINANTLY ANTIBODY DEFICIENCIES

Predominantly antibody deficiencies are 1 of the most commonly diagnosed PIDs, with a clinical presentation that includes increased susceptibility to infections, autoinflammatory disorders, and autoimmune diseases [42]. The onset of PADs is diverse, potentially arising from defects in early B-cell development, immunoglobulin (Ig) class-switch recombination, or terminal B-cell differentiation [43]; disease severity for PADs can be loosely classified as mild (IgG subclass deficiency, specific antibody deficiency, or primary hypogammaglobulinemia), moderate (uncomplicated common variable immunodeficiency disorders [CVIDs]), or severe (complicated PAD, often with an underlying monogenic cause) [30]. In populations with PADs, the most frequently diagnosed symptomatic PID is the CVID phenotype [44]. Although the exact genetic defect underlying CVID is unknown, it is generally identified by the reduction in at least 2 serum Ig isotypes and, in some cases, defects in T-cell immunity, consequently leading to increased susceptibility to infection [2, 45].

Because antibody responses are crucial to limit SARS-CoV-2 infections, patients with PADs remain at increased risk of severe COVID-19, with a 2.3-times higher risk of infection requiring hospitalization compared with the general population [46]. Given the variable underlying causes of PADs, SARS-CoV-2 infection rates vary depending on disease type (Table 1). Specifically, studies have reported SARS-CoV-2 infection rates ranging from 0.2% to 69.6% for CVID and 0.6% to 85.7% [11, 13, 14, 34] for congenital agammaglobulinemia (characterized with low levels or complete absence of B cells, leading to severe reduction in all serum Igs) (Table 1) [11, 13, 16, 47]. In addition, patients with PADs may experience a longer COVID-19 disease course and prolonged SARS-CoV-2 shedding compared with populations without PADs [46, 48]. Consequently, a high proportion of patients with PADs experience severe COVID-19 outcomes, with 56.5% of patients with CVIDs and 79% of patients with congenital agammaglobulinemia requiring hospitalization [14, 16]. In addition, case-fatality rates of up to 61.5% [11, 13, 14] and 7.2% [11, 13, 16, 47] have been reported for patients with CVID and congenital agammaglobulinemia, respectively.

In populations with PADs, the response to COVID-19 vaccination has been shown to correlate with disease severity and clinical complications [28, 30]. Patients with CVIDs had binding antibody seroresponse rates between 14.3% and 100% after a primary COVID-19 vaccination, albeit with lower antibody concentrations compared with healthy controls [7, 9, 21, 24–38]; it is notable that a few studies reported seroresponse rates below 50% [9, 24, 25, 27, 29, 34–38]. Neutralizing antibodies were detected in 50% to 80% of individuals with CVIDs who completed a 2-dose primary COVID-19 vaccine series (Table 2) [9, 24, 26, 27, 29, 30, 35]. Cellular responses were generally induced in 67% to 83% of patients with CVIDs after COVID-19 vaccination, with only 1 small study among patients with CVIDs vaccinated with BNT162b2 reporting that no patients developed cellular immune responses [7, 24, 26, 28, 29, 34]. In addition, another study reported that patients with CVIDs vaccinated with 2 doses of mRNA-1273 mounted comparable T-cell levels to healthy controls [24]. Furthermore, when patients with CVIDs were administered an additional COVID-19 vaccine dose, 3 of 4 studies found a significant increase in antibody responses, supporting the use of a 3-dose primary series to improve humoral responses to vaccination in this population [24, 27, 30, 37].

Antibody responses after COVID-19 vaccination varied greatly in patients with congenital agammaglobulinemia, with almost half of the studies included in this review (4 of 9) reporting seroconversion rates of 0% [27, 36]. These findings are unsurprising given the severity of immunodeficiency in these patients, resulting in poorer vaccine-mediated responses than in patients with CVID [2]. However, 75.0% to 100% of patients with congenital agammaglobulinemia did develop T-cell responses in 1 study [27].

Guidance on whether individuals require a COVID-19 a 3-dose primary series can be extrapolated from their vaccination history (COVID-19 and non-COVID-19) as well as their seroresponse rates after vaccination. Such data can guide the administration of additional vaccine doses and/or alternative therapy, such as Ig replacement therapy (IGRT), to sufficiently protect populations with PADs [49]. Because certain individuals with PADs cannot mount protective humoral responses, therapies such as IGRT can assist in protecting these populations against SARS-CoV-2 and circulating variants [9]. These therapies aid in bolstering immune responses in these individuals, with evidence suggesting that IGRT can be administered concomitantly with vaccination without negatively impacting vaccine responses [9, 20, 29].

In summary, patients with PADs are at an increased risk of severe COVID-19 and death. Although patients with PADs generally mount poor humoral responses to COVID-19 vaccination compared with healthy populations or populations with CIDs, limited evidence indicates that cellular responses may provide some protection against severe COVID-19 and death in this population.

## POPULATIONS WITH COMBINED IMMUNODEFICIENCIES

Primary immunodeficiency populations with CIDs are characterized by both humoral and cellular deficiencies, and, consequently, it is unsurprising that they experience high hospitalization and mortality rates after COVID-19 (Table 1) [2, 50]. Although SARS-CoV-2 infection rates among PID populations with CIDs with associated or syndromic features appears to be low, ranging from 0.0% to 7.7%, the range of COVID-19-related hospitalization in these populations is substantially wider [51], with reports from rates as low as 0.4% to as high as 37.9% [12, 15]. It is interesting to note that in some CID populations, such as those with nonsyndromic CIDs, mortality rates as high as 83.3% have been reported [11], whereas in others, such as those with CD4 lymphopenia, ataxia telangiectasia, and hyper IgE syndrome, no COVID-19-related mortality was reported, although this observation may be attributed to the small sample size of the study [11, 13].

Despite the high disease burden, COVID-19 vaccination is expected to provide some level of protection from severe disease in these patients (Table 2). Specifically, a study reported that patients with CIDs develop antibody and cellular responses after COVID-19 vaccination that are comparable to those in individuals without CIDs [26]. Furthermore, although there is evidence to show that some patients with CIDs are unable to mount humoral responses to vaccination, studies including larger sample sizes report that as many as 71.0% to 90.9% of patients with CIDs mount binding antibody responses after a 2-dose primary series mRNA COVID-19 vaccination [26] and, in addition, that these responses can be enhanced with the receipt of an additional dose of mRNA COVID-19 vaccine [24].

Taken together, these studies suggest that although patients with CIDs are at increased risk of developing severe COVID-19, they can mount potentially protective immune responses after vaccination that can be further increased by booster vaccination. Although the large range of vaccination response rates may be attributable to the variation among underlying conditions and small sample sizes of the studies summarized, additional studies are needed to confirm clinical effectiveness [24].

## COVID-19 VACCINATION RECOMMENDATIONS FOR POPULATIONS WITH PRIMARY IMMUNODEFICIENCIES

The Centers for Disease Control and Prevention (CDC) and the World Health Organization both recommend that people with PIDs receive an initial 2-dose mRNA COVID-19 vaccination series (Table 3), followed by an additional mRNA COVID-19 vaccine dose between 2 and 3 months after dose 2 [52, 53]. In addition, for optimal protection, the CDC also recommends the administration of a bivalent mRNA-based booster dose (≥2 months after the third dose) in all immunocompromised populations aged 5 and older [52]. Such recommendations highlight the importance of ongoing vaccination programs to provide continual protection from COVID-19 in populations with PIDs.

**Table 3.** mRNA COVID-19 Vaccination Recommendations for Patients With PIDs

Institute/Organization	Guidance Document	Considerations/Recommendations
Centers for Disease Control and Prevention [52]	COVID-19 vaccines for people who are moderately or severely immunocompromised	Aged 6 Months to 4 Years A 2-dose primary series of mRNA vaccine should be administered An additional dose should be administered ≥8 weeks postdose 2 Aged 5 to 11 Years A 2-dose primary series of mRNA vaccine should be administered An additional dose should be administered ≥4 weeks postdose 2 An mRNA-based booster dose should be administered ≥2 months after dose 3 Aged 12 to 17 years Administration of a 2-dose mRNA vaccine primary series An additional dose should be administered ≥4 weeks postdose 2 for those who received an mRNA-based primary series An mRNA-based booster dose should be administered ≥2 months after the final dose regardless of primary vaccine series Aged ≥18 Years Administration of a 2-dose mRNA or Novavax vaccine, or 1-dose J&J/Janssen vaccine primary series An mRNA-based additional dose should be administered ≥4 weeks postdose 1 (J&J/Janssen) or dose 2 (mRNA vaccine) An mRNA-based booster dose should be administered ≥2 months after the final dose regardless of primary vaccine series
World Health Organization [53]	Interim recommendations for an extended primary series with an additional vaccine dose for COVID-19 vaccination in immunocompromised persons	An additional mRNA vaccine dose should be administered 1 to 3 months after the primary series

Abbreviations: PID, primary immunodeficiency.

## CONCLUSIONS

Patients with PIDs are at higher risk of severe outcomes after SARS-CoV-2 infection than the general population due to disease-specific deficiencies that may prevent them from mounting sufficiently protective immune responses. Much of the available literature on outcomes of SARS-CoV-2 infection and vaccination in populations with PIDs focuses on patients with CIDs or PADs, with limited to no information available on other PID populations. Patients with CIDs and PADs both face a high burden of disease and mortality after SARS-CoV-2 infection. Studies of monoclonal antibodies among patients with PID are an emerging area of potential protection from SARS-CoV-2 infection [54–56]. Given the resistance elicited to the currently circulating omicron strain [54], further evaluation of the potential therapeutic use of monoclonal antibodies is warranted. Despite variable responses to vaccination, accumulating evidence suggests that COVID-19 vaccination may elicit humoral and/or cellular responses depending on the PID, albeit the breadth, quantity, and quality of these responses have not been uniformly assessed in this population. Standardized immunogenicity assessment and an established correlate of protection are needed and would greatly aid in determining the level of adequate protection from severe outcomes in this heterogeneous population. Effectiveness studies assessing outcomes after COVID-19 vaccination are greatly needed to better understand the impact of current vaccination recommendations on the burden of COVID-19 disease in patients with PIDs and guide the frequency and timing of booster doses and other therapies. In addition, studies are needed to assess similar outcomes in other PID populations, which will better inform vaccination policies with evidence-based recommendations to maximize protection among this highly vulnerable population and ensure that it is protected from COVID-19.

## Notes

***Acknowledgments*.** Medical writing and editorial assistance were provided by Wynand van Losenoord and Jared Mackenzie, PhD (MEDiSTRAVA), in accordance with Good Publication Practice (GPP 2022) guidelines, funded by Moderna, Inc., and under the direction of the author.

***Supplement sponsorship.*** This article appears as part of the supplement “COVID-19 Vaccination in the Immunocompromised Population,” sponsored by Moderna, Inc.
